# Editorial: Anthropogenic effects on the microbial communities of terrestrial ecosystems

**DOI:** 10.3389/fmicb.2025.1636546

**Published:** 2025-07-16

**Authors:** Wenchen Song, Xingjia Xiang, Jianming Wang, Mark Radosevich, Yaping Lin

**Affiliations:** ^1^College of Life and Environmental Sciences, Minzu University of China, Beijing, China; ^2^Department of Ecological Sciences, Anhui University, Hefei, China; ^3^College of Ecology and Nature Conservation, Beijing Forestry University, Beijing, China; ^4^Department of Biosystems Engineering and Soil Science, The University of Tennessee Knoxville, Knoxville, TN, United States

**Keywords:** anthropogenic effects, terrestrial microbial communities, agricultural microbial communities, grassland management on microbial communities, forest management on microbial communities, pollution on microbial communities, urbanization on microbial communities

## Introduction

Microorganisms in terrestrial ecosystems, including bacteria, fungi, archaea, protozoa, and microalgae (Chen et al., 2024), are one of the vital components of terrestrial ecosystems. Terrestrial microbial communities act as the core drivers of biogeochemical cycles and are the supporters of the productivity and stability of terrestrial ecosystems. They maintain the material cycles and energy flows of terrestrial ecosystems with their species diversity, genetic diversity, functional diversity, and complex metabolic networks (Song, 2023, 2024; Chen et al., 2024; Hu et al., 2024; Zhao et al., 2025; Srivastava et al., 2025; Steinauer et al., 2023). However, with the intensification of industrialization and modernization in various countries, the negative impacts of human activities on terrestrial ecosystems have gradually become apparent, and scholars have increasingly focused on the effects of anthropogenic factors on microbial communities in terrestrial ecosystems (Maehara et al., 2024; Philippot et al., 2021; Xue et al., 2024). Compared with natural factors, anthropogenic factors are more urgent due to their complexity and potential risks that have not yet been fully recognized. The ability of terrestrial ecosystems to resist anthropogenic disturbances and maintain their ecological functions is being increasingly challenged. Therefore, it is particularly important to explore the impact of anthropogenic factors on microbial communities in terrestrial ecosystems.

**Figure 1:**
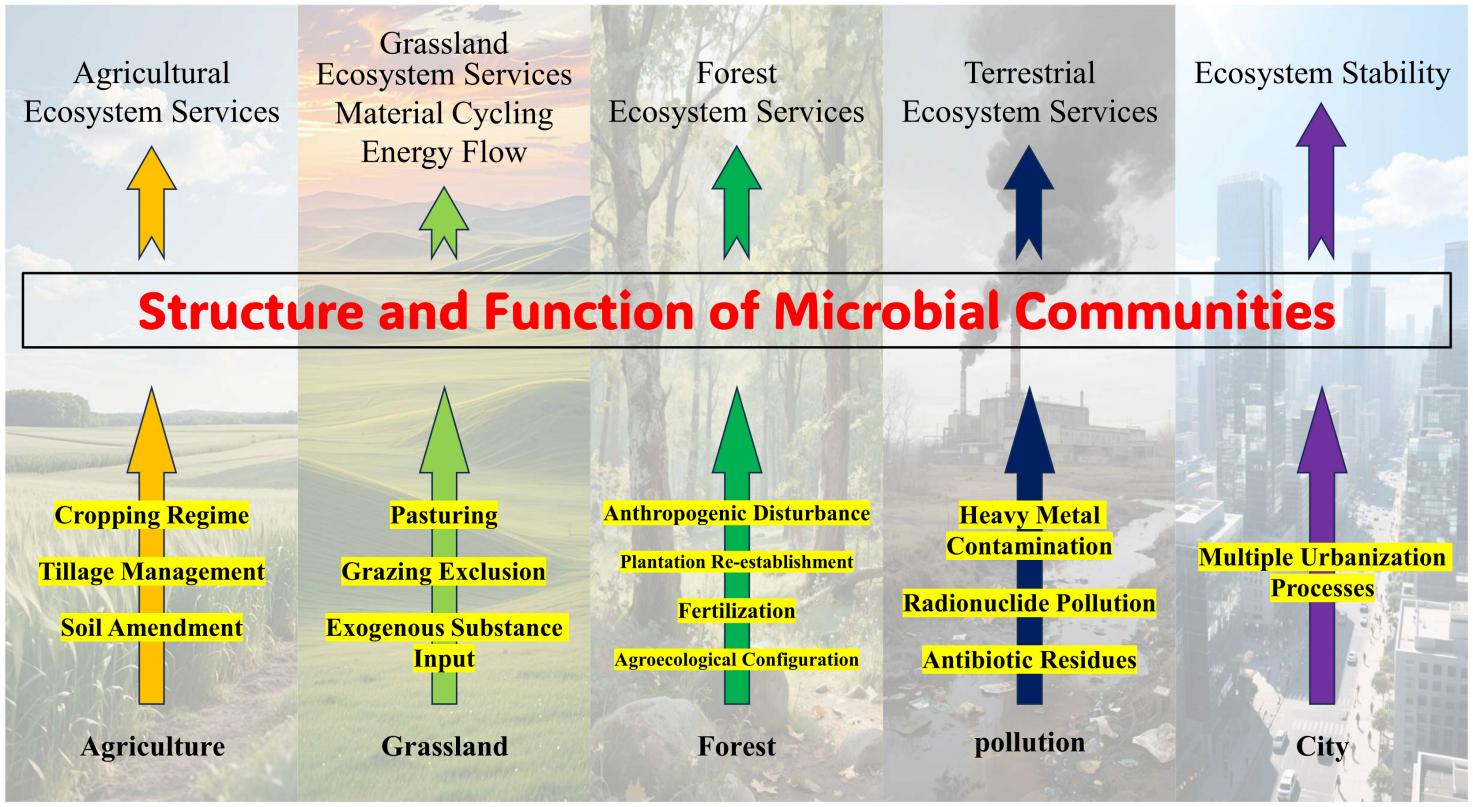
Schematic diagram of the impact of anthropogenic factors on microbial communities in terrestrial ecosystems.

With the discovery and application of new technologies such as microbial genomics, transcriptomics, and DNA-SIP, we are now more capable than ever of revealing and improving the impact of anthropogenic factors on the structure and function of microbial communities in terrestrial ecosystems. Thus, this Research Topic in Frontiers in Microbiology aims to systematically analyze the impact mechanisms of anthropogenic factors on microbial communities in terrestrial ecosystems from aspects such as agricultural activities, grassland management, forest management, pollution, and urbanization (Figure 1). This Research Topic was a success since 35 Articles were collected here and called on scholars worldwide to actively participate in related research.

## The impact of agricultural activities on microbial communities

Modern agricultural activities play a significant role in altering the structure and function of microbial communities in terrestrial ecosystems. This Research Topic focuses on how different cropping patterns, tillage management, and soil amendments in agricultural activities can regulate microbial communities to enhance ecosystem services in agriculture.

Cropping patterns mainly include monoculture, crop rotation, and intercropping. Song et al. and Ding et al. who studied the soil of continuously cropped wine grapes in arid regions and the rhizosphere soil of tobacco that had been monocropped for many years, respectively, found that long-term monoculture significantly reduces soil enzyme activity, alters microbial community structure, and disrupts nutrient cycling functions. In contrast, Liu L. et al., Yan et al., and Wang B. et al. analyzed the changes in soil microbial community structure and function under different crop rotation patterns, such as tobacco-oilseed rape rotation and tobacco-maize rotation, and discovered that crop rotation significantly affects soil microbial diversity and composition, influencing the health and productivity of agricultural ecosystems. Meanwhile, Liu J. et al. who studied the soil of poplar and soybean intercropping, found that intercropping can change the composition of soil microbial communities and improve soil physicochemical properties such as phosphorus content. Both crop rotation and intercropping can to some extent offset the negative impacts of long-term monoculture on soil physicochemical properties and the structure and function of microbial communities.

Tillage management is an important aspect of agricultural production. This Research Topic explored the impact of different agronomic practices on soil microbial community diversity. The articles by Li H. et al., Jia et al., Guo et al. and Wei et al. found that rational agronomic measures can significantly enhance soil microbial community diversity, thereby improving soil health and promoting the recovery of agricultural ecosystems. Li H. et al. investigated the effects of different straw cover frequencies and amounts on the structure and function of rhizosphere and endophytic microbial communities in crops and found that low-frequency, high-coverage straw can significantly reduce the abundance of pathogenic bacteria while increasing the proportion of nitrogen-fixing bacteria. Moderate coverage can promote the complexity and optimization of microbial community structure and function. Jia et al. found that long-term nitrogen fertilization and straw incorporation significantly increase N_2_O emissions and potential nitrification rates (PNR), thereby affecting soil microbial activity and soil nitrogen cycling. Guo et al. discovered that a combination of 600,000 plants per hectare, 1,800 m^3^ of irrigation per hectare, and 150 kg of urea per hectare in the Aksu region can maintain high microbial diversity and soil health. Wei et al. found that composting can improve soil conditions and regulate rhizosphere microbial communities, thereby increasing the productivity of alfalfa in saline-alkali soils and providing an effective strategy for the ecological restoration of saline-alkali lands.

Good soil conditions are the foundation for stable and high agricultural production. In this Research Topic, Ye et al. and Xiao et al. studied the relationship between soil microbial community diversity and soil functional diversity under various human interventions, including biochar addition. The results indicate that appropriate human interventions can significantly enhance soil microbial community function and promote the availability of soil nutrients, thereby improving the growth environment for crops.

## The impact of grassland management on microbial communities

Grassland ecosystems are an important component of terrestrial ecosystems, and the microbial communities within them play an irreplaceable role in material cycling, energy flow, and ecosystem services. Rui et al. revealed the distribution dynamics of prokaryotic communities in the soil of the Tibetan Plateau alpine grasslands along altitude and seasonal gradients, emphasizing the key role of moisture conditions in regulating community assembly processes. Grassland management is crucial for maintaining the stability of grassland ecosystems. This Research Topic, combined with the latest research findings of many scholars, explores the impact of different grassland management strategies on the structure and function of grassland microbial communities.

Overgrazing is one of the important anthropogenic drivers of grassland ecosystem degradation. Fang et al. (a) using the Hulunbuir grassland in Inner Mongolia as a study site, explored how overgrazing on unmanaged grasslands affects the recovery of adjacent grazing-banned grasslands and found that overgrazing on unmanaged grasslands has a negative impact on the ecological recovery of adjacent grazing-banned grasslands, manifested in the deterioration of soil properties, simplification of plant communities, and singularization of microbial community functions in both the unmanaged grasslands themselves and the grazing-banned areas close to them.

For a long time, grazing bans have been considered an important means of ecological restoration in grassland ecosystems. However, Jiang et al. by studying three different types of grasslands (temperate desert, temperate grassland, and mountain meadow), explored the impact of long-term grazing bans on soil nutrients and fungal community structure and found that the effect of grazing bans varies with grassland type. The fungal network modularity is the highest in the grazing-banned areas of temperate grasslands, with significant enhancement of community stability, while in the grazing-banned areas of mountain meadows, soil nutrient levels decline, and grazing bans exacerbate nutrient imbalances.

In addition, this Research Topic also explored whether the addition of exogenous substances to grassland ecosystems would lead to soil carbon emissions driven by microbial communities. Liu G. et al. indicate that short-term addition of exogenous substances only causes weak decomposition of soil organic matter, while long-term addition promotes carbon sequestration through the microbial carbon pump mechanism rather than continuous emissions. This has positive implications for maintaining the stability of soil carbon stocks in grassland ecosystems and addressing climate change.

## The impact of forest management on microbial communities

Forests are a core component of terrestrial ecosystems, and the microbial communities within forest ecosystems are the main drivers of element cycling and vegetation renewal. These communities are also influenced by natural factors. Lian et al. found that light and moisture significantly affect the rhizosphere microecology of two types of oak seedlings, influencing soil enzyme activity and microbial community structure.

However, forest ecosystems are currently facing unprecedented human disturbances, among which anthropogenic wildfires and logging are inevitable interferences in forest succession. These two types of human-induced forest disturbances alter the dynamics of microbial communities in terrestrial ecosystems through different mechanisms, thereby affecting the structure and function of forest ecosystems.

Liu G. et al. using Chinese pine forests in North China as a study object, investigated the impact of wildfire intensity and soil stratification on soil physicochemical properties and analyzed changes in microbial community diversity and composition through 16S rRNA sequencing. They found that high-intensity wildfires significantly increase soil pH, reduce inorganic phosphorus content and nitrate nitrogen supply rates, leading to a decrease in the abundance of ectomycorrhizal fungi and an increase in the proportion of pathogenic bacteria. Robinson et al. studying the effects of selective logging on soil microbial communities and their functions in Borneo's tropical forests, found that selective logging weakens soil ecosystem services by altering key microbial communities. Specifically, selective logging significantly reduces the abundance of ectomycorrhizal fungi in soil microbial communities, increases the abundance of arbuscular mycorrhizal fungi, leads to a decline in soil inorganic phosphorus concentration and nitrate supply rates, and reduces the trend of soil heterotrophic respiration.

In the face of frequent human disturbances, sustainable forest management measures such as reforestation, fertilization, and optimization of planting patterns have emerged. These measures can regulate the ecological service functions of forest ecosystems by altering vegetation structure, soil environment, and the structure and function of microbial communities. Wang C. et al. revealed that in poplar plantations, the dynamic assembly of bacterial communities is mainly driven by stochastic processes and is significantly influenced by environmental factors such as temperature and precipitation. They also emphasized the role of key species in maintaining community stability. Hou et al. studying a man-made fir forest in South China, explored the impact of reforestation on the structure and function of soil prokaryotic microbial communities and found that reforestation can change soil microenvironments, select microbial taxa adapted to specific environments, and affect carbon and nitrogen cycling efficiency. Qin et al. comparing phosphorus cycling genes in different soil layers of three forest types, found that converting a mason pine plantation to a broad-leaved forest significantly enhances the phosphorus-solubilizing potential of surface soil microorganisms, while converting it to a mixed forest increases the structural stability of the phosphorus cycling gene network, making it more resilient to environmental changes. Li D. et al. studying the interaction between plants and their rhizosphere bacterial communities at different successional stages of a subtropical secondary forest, found that organic fertilizers, compared to inorganic fertilizers, significantly increase bacterial diversity and promote species growth, thereby facilitating positive forest succession. Tian et al. discovered the response of soil microbial communities to forest succession and the root condition of specific tree species in a subtropical pine-oak mixed forest. However, it is important to note that some management measures may pose risks. Tavares et al. found that converting forests to pastures changes soil microbial composition and may increase the environmental spread of antibiotic resistance genes through management practices such as fertilization.

## The impact of pollution on microbial communities

Since the advent of the industrial civilization era, frequent industrial construction and other human activities have given rise to a variety of environmental pollutants. These pollutants, once released into the environment, are altering microbial communities in terrestrial ecosystems. This Research Topic, in combination with the latest research findings from various scholars, reveals the impact of pollutants on microbial communities in terrestrial ecosystems and the mechanisms by which microbial communities respond to pollutants.

Heavy metal pollution is severely threatening and reshaping the structure and function of soil microbial communities. Liu H. et al. and Zhang X. et al. investigated the complex effects of cadmium (Cd) and chromium (Cr) pollution on soil microorganisms and found that heavy metal pollution can alter soil physicochemical properties, thereby directly or indirectly disrupting the structure of microbial communities and reducing their ecological stability. Liu H. et al. also discovered that the microporous structure of biochar can adsorb heavy metals, while organic manure can reshape microbial structure through carbon and nitrogen supply. The combined application of the two can positively improve soil conditions affected by heavy metal pollution and enhance crop quality, providing a green solution for the remediation of heavy metal-contaminated land.

With the continuous development of nuclear industry and nuclear science in various countries, an increasing number of ecosystems are being exposed to ionizing radiation pollution over the long term. Zeng et al. through a study of plant-microbe-soil ecosystems exposed to low-level radiation (LLR) for 10 years, analyzed the changes in radiation absorption dose rates with distance and found that LLR affects the carbon and nitrogen migration processes between plants, microbes, and soil through symbiotic microbial effects. They also showed that increased radiation intensity leads to a significant increase in the relative abundance of symbiotic fungi, accompanied by increases in soil lignin peroxidase activity, C/N ratio, and carbon content, and induces adaptive changes in plant functional traits.

On a global scale, Wepking et al. through a study of the residual effects of antibiotic use on the temperature response of soil microorganisms, found that compared with non-polluted soil, soil samples contaminated with antibiotics exhibit different evolutionary trajectories of microbial community structure and function under warming conditions, which may greatly disrupt the stability of soil carbon cycling and, in turn, affect the accuracy of global carbon budget predictions. Yang et al. found that plastic pollution has become a global environmental issue, posing potential ecological risks to microbial communities in soil ecosystems. However, there are still gaps in the long-term ecological impact of plastic pollution on soil microbial communities and the combined effects of plastic with other pollutants. Future research needs to be conducted over a wider range of environmental conditions for a comprehensive assessment of the ecological risks of plastic pollution.

Wu et al. focusing on ecological restoration, discovered the potential of phosphate-solubilizing bacteria (PSB) in high-altitude harsh environments, providing a sustainable solution for the ecological restoration of the Mu Li coal mine area while reducing dependence on chemical fertilizers and lowering environmental pollution risks.

## The impact of urbanization on microbial communities

Urban areas represent highly anthropogenic ecosystems, and their influence on microbial communities in terrestrial ecosystems is increasingly becoming a focal point of current research. From the green space soils in Jiangnan cities to the urban fringes of northern grasslands, this Research Topic integrates the latest findings to reveal how multiple urbanization processes alter the structure and function of microbial communities, thereby threatening ecosystem stability.

Dai et al. in their study of soil microbial communities in urban and rural citrus orchards in Zhejiang Province, found that in urban soils, the abundance of heavy metal-tolerant bacteria such as Bacillus significantly increased. The over proliferation of such bacterial groups, coupled with the decline of beneficial bacteria, led to an increased risk of root rot, indicating poor disease regulation capacity in urban ecosystems. Zhang F. et al. focused on the urbanized areas of Nanchang City, examining soil aggregates under different urbanization intensity gradients. By studying how soil microbes and enzymes influence the dynamics of soil organic carbon (SOC) during urbanization, they discovered that as urbanization intensity increased, both SOC content and storage significantly decreased, along with microbial and enzyme activity. Structural equation modeling indicated that urbanization primarily reduced SOC by lowering the total nitrogen (TN) and total phosphorus (TP) content in the soil, which in turn suppressed microbial biomass and led to a decline in carbon sequestration capacity.

Vegetation is an essential component of urban ecosystems and often serves as a refuge for microbial communities alongside soil. Fang et al. (b) using the Hulun Buir grassland in Inner Mongolia as a study site, explored the impact of pastoral town construction on adjacent grassland ecosystems. They found that the soil in grasslands within 1 km of urban areas, compared to those 3 km away, experienced a decrease in total organic carbon (TOC) and soil water content (SWC), which weakened the decomposition function of lignicolous fungi. Meanwhile, increases in pH and TP promoted the proliferation of pathogenic fungi. The rise in pathogenic fungi led to a decline in plant diversity, further reducing litter input and exacerbating nutrient cycling obstacles in the soil. In other words, urbanization often comes at the cost of disrupting the interactions between plants and soil microbial communities, leading to the degradation of adjacent grassland ecosystems. Li T. et al. discovered that photovoltaic power stations can significantly affect the structure and network characteristics of microbial communities by altering soil properties. In response to these changes, bacterial communities are more sensitive, while fungal communities exhibit stronger adaptability.

## Conclusions and future directions

This Research Topic collects 35 articles from scholars from different parts by systematically reviewing the impact of various typical human activities, such as agricultural practices, grassland management, forest management, pollution, and urbanization, on the structure and function of microbial communities in terrestrial ecosystems, it has revealed that human factors primarily alter the ecological niches of microbial communities by directly changing the soil environment or plant growth conditions on which microbial communities depend. This ultimately affects the stability of terrestrial ecosystems and provides a scientific basis for understanding the vulnerability of microbial communities and ecological restoration.

Despite the significant progress made in current research, there are still limitations. For example, studies often focus on a single disturbance factor, while terrestrial ecosystems are actually frequently subjected to multiple pressures from various sources. The depth of functional analysis of microbial communities is insufficient, and there is a lack of mechanistic explanations for the specific environmental adaptation strategies of microbial groups. Most existing studies are based on the analysis of microbial communities using 16S rRNA gene sequencing results, lacking research on the metabolic functions of microbial communities. Future research directions should focus on integrating studies from multiple factors and perspectives, combining techniques such as metagenomics or metabolomics to reveal changes in the structure and function of microbial communities. It is also possible to investigate the long-term evolutionary patterns of microbial communities at observation stations in the studied ecosystems. There is a call for interdisciplinary cooperation among experts and scholars in related fields to conduct joint research.
